# Study on Epoxy Resin Composite Reinforced with Rice Straw Fiber

**DOI:** 10.3390/ma16041370

**Published:** 2023-02-06

**Authors:** Xinzhen Liu, Jiaxin Wang, Tianqi Liu, Qian Cheng, Anhui Li, Yuge Li, Zihui Liu, Jiayi Sun, Dejun Liu

**Affiliations:** College of Engineering, Shenyang Agricultural University, Shenyang 110866, China

**Keywords:** rice straw, fiber, composite material, pretreatment, epoxy resin

## Abstract

In order to enhance the performance of the epoxy resin-prepared materials, straw fiber was used as the reinforcing base in this study. The principle of this study is to use the cellulose component exposed after the defibrillation of straw fiber can be further combined with the epoxy group. Firstly, the degree of defibrillation of straw fiber under three different pretreatment methods of acid, alkali and moist heat treatment was explored, and a control test was conducted with untreated straw fiber, which showed that the defibrillation of the straw fiber after alkali treatment was better than the other two methods. Secondly, to prove the comprehensive effect of the pretreatment method and straw fiber filling amount on the composite material performance, this paper carried out a tensile, bending, density and water absorption test. The results showed that when the straw fiber filling was 15%, the best performance of the composites was achieved by the alkali treatment, with tensile strength and tensile modulus reaching 1.89 KN and 3.92 MPa, bending strength and bending modulus reaching 2.00 KN and 81.65 MPa, average water absorption reaching 2.77%, and density reaching 0.957 g/cm^3^. Finally, the results were verified using Image J software was used for verification. After comparison, the material meets the basic requirements of high-density fiberboard material and provides a reference for preparing straw epoxy resin composites.

## 1. Introduction

Epoxy resin materials have excellent physical and electrical insulation properties and can bond with various materials [1,2]. Compared with traditional resin materials, the preparation of epoxy resin is easier, energy saving and non-toxic. Compared with other thermosetting resins, the epoxy resin process’s flexible performance has not been provided by other materials [3,4,5]. Due to its poor performance, it also has certain limitations, such as high brittleness.

To solve these problems, scholars have used different materials as reinforcement, such as carbon fiber, glass fiber, graphene, nano-clay, and aluminum metal to improve the properties of epoxy resin materials [6,7,8,9,10]. However, these fibers are difficult to obtain, expensive to produce, and consume a lot of energy. Compared with these fibers, plant fiber is not only easy to get, and the process is simple, but also has the advantages of large reserves, recycling, and regeneration. In recent years, with the continuous development of straw-plastic composites, some plant fibers with good mechanical properties such as rape, palm leaf, sisal, jute, cotton fiber, and ramie have also been prepared into composites especially epoxy resin composites [11,12,13,14,15,16]. Nevertheless, the primary use of these fibers is for reinforcement, so the regular use of small, mainly for a variety of fiber mixed-use, single use of fiber is not much, rarely involved in the application of large dosage. In addition, some studies also focus on agricultural and forestry wastes, such as bamboo, poplar, walnut shell, corn, and reed as reinforcing components of epoxy resin composites [17,18,19].

Qi Xianchao et al. [17] prepared composites from long bamboo fibers with epoxy resin and curing agent at a hot pressing pressure of 1.6 MPa, temperature up to 90 °C, and a hot pressing time of 20 min. Furthermore, evaluated the mechanical properties of the composites and found that the tensile strength, tensile modulus, flexural strength, and bending modulus of the composites under the action of long bamboo fibers reached 184 MPa, 6912 MPa, 273 MPa, and 30.824 MPa, respectively. It was also found that the pretreated fibers exhibited better properties. A.B.M. Supian et al. [18] investigated the physical and mechanical properties of date palm fiber reinforced epoxy resin composites, composites obtained by hybridization of date palm and bamboo, which were prepared by hand lay-up method. Compared with pure composites after treatment of date palm fibers received composites in all properties were significantly improved, tensile strength, flexural strength and impact strength reached 39.16 MPa, 61.10 MPa, and 12.70 J/m, respectively, fully proving that the fibers can epoxy resin composites physical properties. Additionally, in the water absorption swelling and water absorption tests, it was found that the properties of composites with date/bamboo fiber hybrids were reduced by 27.68% and 15.39, respectively, compared to those of composites with single date fiber. N.M.Z. Nik Baihaqi et al. [19] also explored the effect of blending palm fiber with carbon fiber reinforced epoxy resin composites using the hand lay-up method with palm fiber fillings of 5, 10, 15, and 20 wt% to explore the fiber to epoxy reinforced palm composites. The ratio of matrix to reinforcement was 85:10 and 80:20, the percentage of palm fiber to carbon fiber was set to 50:50 and 60:40, and the composites were tested in bending and torsion. The tests demonstrated that the bending and torsional properties were improved by 7.4% and 75.61%, respectively, when the fiber filling amount reached 15% compared to other fiber-filled composites. It was shown that fibers can enhance the properties of the epoxy resin matrix and that the fiber filling directly affects the properties exhibited by the composites.

Although the price of agricultural straw is low, it has good mechanical properties and suitable materials [20,21,22,23]. However, a small amount of these agricultural wastes is currently used in straw fuel, straw feed, straw composite materials, and straw biomaterials [24,25,26,27]. The treatment of most of the remaining straw is unified incineration and discard, which will not only cause pollution but also reduce soil fertility and impoverish the land [28,29]. Chinese principal straw originates from rice and corn, which can reach 680 million tons a year, including the rice straw accounting for 74%. In addition, as shown in Table 1, the fiber component of rice straw is the highest among different straw components, and the chemical composition of its cell wall is shown in Table 2. It can be seen that the fiber component of the whole stalk is the highest in rice straw [30]. Therefore, to make full use of rice resources and improve the utilization rate of resources, this study will enhance the preparation and performance of epoxy resin composite materials by different pretreatment methods of straw.

In summary, epoxy resin has the characteristics of solid cohesiveness and stable structure, combined with the high cellulose and lignin content of rice straw. The two materials are combined through specific treatment methods to prepare a composite material with good mechanical properties and physical and chemical properties, which can provide a practical reference for rice straw composite material. The primary purpose of this study is to prepare composites of straw fiber and epoxy resin using epoxy resin and agricultural waste straw. In this study, the bending resistance, tensile resistance, water absorption capacity and density of composite materials with filling amounts of 5 wt%, 15 wt%, 25 wt%, 35 wt%, and 45 wt% under different pretreatment conditions were tested and combined with the comparison of pore and pore size, the best pretreatment method and the best straw fiber filling amount were obtained. Image J software was used to analyze the air attack on the surface of the composite material, and the pore distribution on the surface of the composite material was shown by the frequency of wave peaks and troughs. The results can guide the utilization of agricultural wastes such as rice straw and develop new or improved techniques for preparing polymer composites.

## 2. Materials and Methods

### 2.1. Materials

Table 3 and Table 4 show the materials and equipment used in the experiment. The VOC content of the epoxy resin and curing agent was 0%, and the configuration ratio was 100:85. The type of rice straw used in the experiment was Liaohe No.3. Meoh and Oxalic acid were all pure. Straw Silk Kneading Machine and Straw Fiber Crusher were developed and produced by Shenyang Agricultural University, Liaoning Province, China. The size of the silica gel mold used in the test was 200 × 60 × 30 mm.

### 2.2. Preparation of Straw Fiber

#### 2.2.1. Straw Fiber Harvest

**Figure 1:**
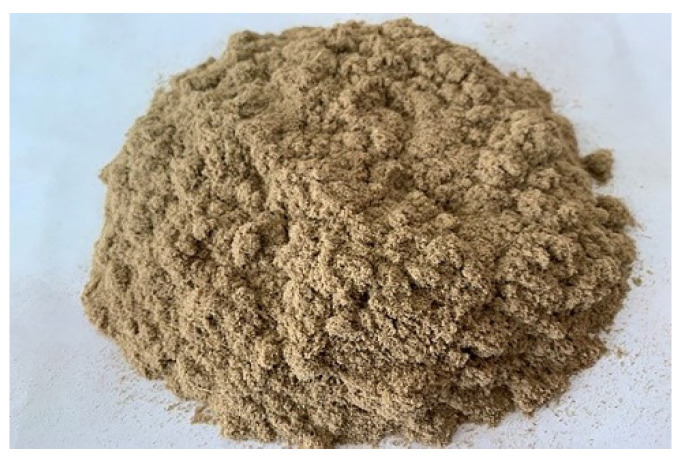
Straw fiber after drying.

The straw treated by the straw kneading machine is sent to the hopper of the straw fiber crusher [31], and the material can be transported to the crushing device stably and continuously through the spiral feeding shaft. The friction and cutting force are applied to the straw through the gap of two grinding wheels to achieve the wax tearing and crushing of the straw fiber surface. The morphological structure of the straw was destroyed, and the straw material was disintegrated. The destruction of the morphological structure of the straw and the disintegration of the straw material led to the generation of individual fibers and the production of high-quality fibers from the whole straw. As the waxy layer of the straw is crushed, so is the waxy layer of the straw. The waxy layer of straw is crushed by surface friction and pressure [32]. This results in increased mixing of the individual fibers with the binder due to the temperature generated during friction, which reduces the moisture content of the straw fibers. Finally, wet straw fiber is obtained, and the fiber is put into the far-infrared drying oven to dry at 60 °C for 6–7 h, as shown in Figure 1, for later use.

#### 2.2.2. Straw Fiber Pretreatment

**Figure 2:**
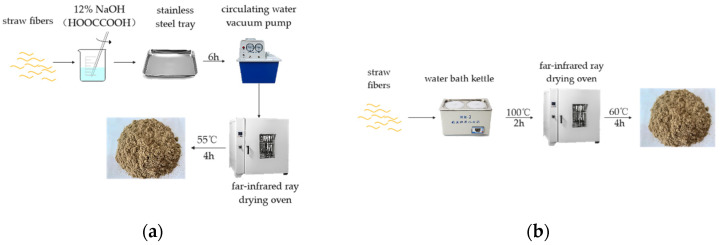
Flow chart of straw fiber pretreatment (**a**) alkali (acid) treatment; (**b**) wet and heat treatment.

Figure 2 is the flow chart of straw fiber pretreatment, Figure 2a is the alkali (acid) treatment flow chart, and Figure 2b is the wet and heat treatment flow chart. The purpose of pretreatment is to remove the wax and ash on the surface of straw and to expose more fiber components, especially cellulose. In the process of composite preparation, the bonding force between straw fibers and the interface binding ability between epoxy groups are the standards to measure the material’s physical properties. Therefore, selecting the most suitable pretreatment method plays an essential role in the performance of composite materials. To explore the pretreatment method, the effects of acid treatment, alkali treatment and wet heat treatment on fiber were analyzed in this study [33]. The specific steps are as follows:

Alkali treatment: Put dry straw fiber in the content of 12% NaOH solution, make the solution contact with straw fiber ultimately, 6 h after immersing in 304 stainless steel tray, the use of circulating water vacuum pump for the suction filter, to be washed to neutral (pH = 7), 55 °C in the far infrared drying oven drying 4 h, after drying of straw fiber by using the high-speed mill, Pass through 250 μm sieve and reserve as ATRSF.

Acid treatment: Put dry straw fiber in the content of 12% HOOCCOOH solution, make the solution contact with straw fiber ultimately, 6 h after immersing in 304 stainless steel tray, the use of circulating water vacuum pump for the suction filter, to be washed to neutral (pH = 7), 55 °C in the far infrared drying oven drying 4 h, after drying of straw fiber by using the high-speed mill, Pass through 250 μm sieve and reserve as ACTRSF.

Wet heat treatment: Put the dried straw fiber into a water bath pot and boil it at 100 °C for 2 h. After the treatment, wash it to neutral and put it into a drying box to dry it at 60 °C for 4 h. Smash it and sieve it, which is recorded as WHTRSF.

#### 2.2.3. Characterization of Straw Fiber

**Figure 3:**
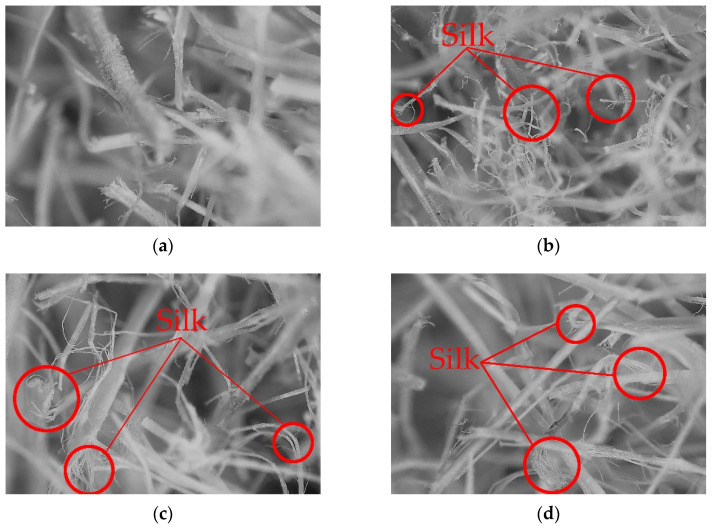
Image of straw fiber pretreatment (**a**) USF; (**b**) ATSF; (**c**) ACTSF; (**d**) WHRSTF.

By the depth of field microscope, the straw fibers were characterized, as shown in Figure 3, as straw fibers under different pretreatment of the microscopic image; the image can be seen in the figure can be seen Figure 3a without pretreatment of straw fiber did not produce filaments, after pretreatment of straw fiber has apparent filaments, Figure 3b shows evident filamentous formation, Figure 3c shows a small amount of filamentous formation, and Figure 3d shows obvious filamentous formation. In particular, straw fiber after alkali treatment showed the best filamentous effect, followed by acid treatment, wet heat treatment, and untreated. Figure 3b clearly shows that the straw fiber after pretreatment shows a large number of holes, indicating that after alkali treatment, a large amount of wax is shed and more fibers are exposed, which is conducive to the complete fusion of the composite material and has enough area to connect with the epoxy resin.

### 2.3. Composite Material Preparation Process

**Figure 4:**
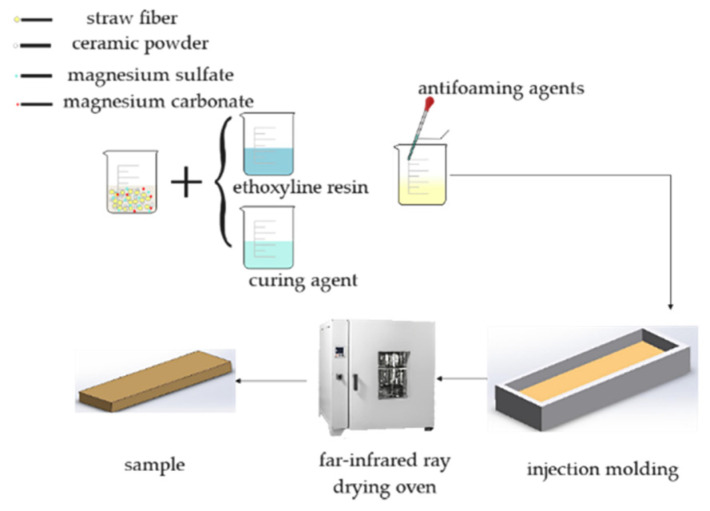
The preparation process of straw composites.

**Figure 5:**
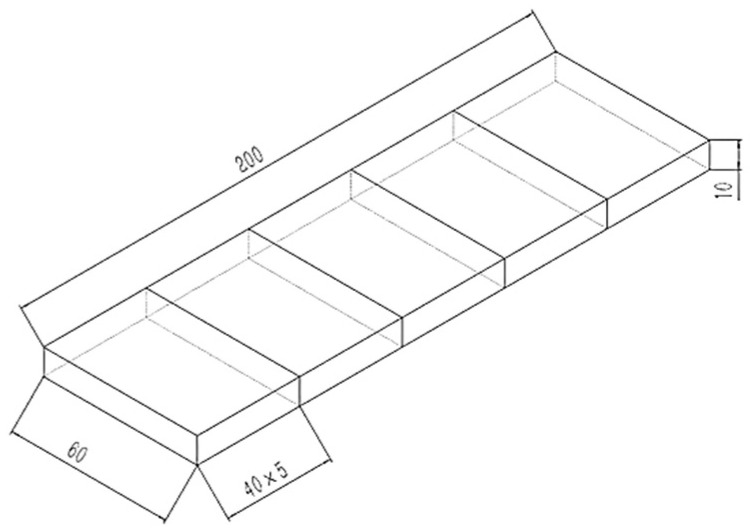
Sample cutting illustration.

The preparation process of straw composite material is shown in Figure 4. The treated straw fiber was soaked in deionized water for 6 h until the fiber was softened and used for future use. In this study, the composite material was prepared by manual layering, and the silicone mold was placed on a flat and smooth table. In order to release the mold, it was easier to apply the release agent inside. A total of 45 g of epoxy resin was added to the beaker, and 15 g of curing agent was added to the beaker. After mixing thoroughly, an appropriate amount of straw fiber was added to the beaker, and the straw fiber was thoroughly stirred with a mixer at 1000 rpm for 3 min. A total of 20 g quartz powder was added in the stirring process to strengthen the surface hardness of the material, and 5 g magnesium carbonate was added to improve the adsorbability of the material. Then, 5 g anti-foaming agent was added to eliminate the internal bubbles generated in the stirring process [34,35]. After stirring for 5 min, the material was transferred into the silicone mold. In order to prevent the internal cavity of the material, a sheet of 100 g weight was placed on the upper layer, and a transparent plastic sheet was placed under the sheet to prevent adhesion. After 16 h of pressing, molding was carried out and placed in a drying oven to dry at 40 °C for 3 h to dry the internal excess water and place it at room temperature. The dried samples were cut, and 3 plates were used for each test, the dimensions of which are shown in Figure 5. The mold was cleaned and the next set of samples were prepared.

### 2.4. Functional Test

#### 2.4.1. Tensile Strength

The tensile strength of the sample was determined according to the tensile test method specified in ASTMD 3039-76 [36]. A universal testing machine was used to test the model at a loading speed of 10 mm/min, and the maximum load was recorded. The tensile properties of the specimen are calculated using Equation (1) [36]:

(1) $$\sigma =\frac{F_{b}}{S_{0}}$$

where *F_b_* is the maximum load and *S*_0_ is the cross-sectional area.

#### 2.4.2. Flexural Strength

The bending strength of all samples was determined according to the three-point bending test method specified in the Chinese national standard GB/T 17657-2013 [37]. A universal testing machine was used to test the samples at a loading rate of 10 mm/min, and the maximum failure load was recorded. The flexural strength of the specimen was calculated using Equation (2) [37]:

(2) $$R=\frac{3PL}{2bh^{2}}$$

where *P* is the maximum load, *L* is the support span, *b* is the width of the specimen, and *h* is the depth of the sample.

**Figure 6:**
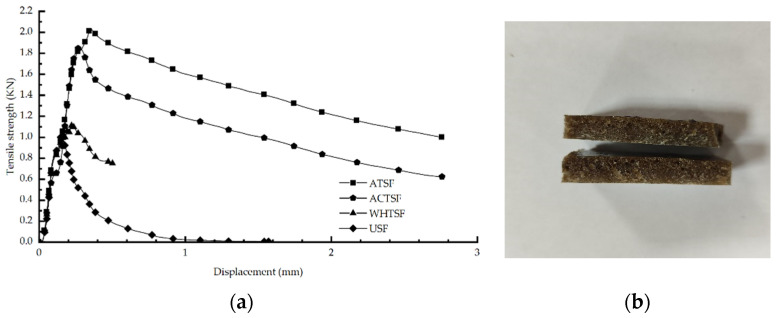
Tensile strength of composite materials: (**a**) the curve of tensile strength; (**b**) composite failure picture.

In the test, the support span L was 200 mm, and the specimen’s width and nominal depth H were 60 mm and 40 mm, respectively, as shown in Figure 6.

#### 2.4.3. Water Absorption Test

By referring to GB/T17657-1999 [38] to test the water absorption rate of the composite material, the sample prepared in Figure 5 was cut along the middle line to obtain a 60 × 40 × 10 mm sample, and its weight was measured as *M*_0_. Samples were dried in an oven at 60 °C, dried to a constant weight, weighed, and measured for thickness, then immersed in a stainless steel tray of deionized water. It was taken out after arrival, the surface moisture was wiped, and it was considered *M*. There were three samples in each group, and the results were presented as mean values. Water absorption is Equation (3) [38]:

(3) $$W=\frac{M-M_{0}}{M_{0}}\times 100\%$$

The mass of the composite material before water absorption is *M*, and the mass after water absorption is *M_0_*.

#### 2.4.4. Density Test

The density of the sample was weighed on the electronic scale by Archimedes method at room temperature and calculated using Equation (4) [39]:

(4) $$\rho =\frac{m_{2}}{m_{2}-m_{1}}$$

where *m*_1_ is the composite material’s weight when it is completely dry; *m*_2_ is the wet weight of the composite after soaking in distilled water.

#### 2.4.5. Surface Aperture Analysis

The straw composite material under different pretreatment methods was analyzed by ImageJ 2 software. The straw composite material was placed under ultra-depth of field microscope of field to take photos and observe the initial Image of the composite material. A 30× microscope was used to observe the straw composite material. After taking pictures, the composite material before and after pretreatment was analyzed by ImageJ software. The Stacks option in Image in the software combines multiple photo groups, and line segments are divided randomly. The data Image is analyzed by Plot Profile in Analyze, and finally the composite aperture map is obtained. The number of pores in the material is indicated by the gray absorption of the pores in the image. The pore size of the composite material is determined according to the different gray values displayed.

## 3. Results and Discussion

### 3.1. Tensile Strength

Table 5 and Table 6 show ANOVA of test results of tensile strength and tensile modulus. It can be seen from ANOVA that the effect of straw fiber filling amount and pretreatment method on tensile strength and tensile modulus is extremely significant, indicating that straw fiber filling amount and pretreatment method have an evident influence on tensile properties.

Figure 6a shows the composite tensile strength versus displacement images, from which it can be seen that the maximum fracture values of the pretreated composites are significantly larger than those of the untreated composites. Moreover, the maximum tensile strength of the alkali-treated composites had the best maximum tensile strength of 2.06 KN. They are followed by the acid-treated composites with a maximum tensile strength of 1.89 KN. Compared with the other two pretreatments, the tensile strength of the moist wet-heat treated composites was not significant with a maximum tensile strength of 1.16 KN. The corresponding tensile stresses of the four pretreatment methods reached 3.92 MPa, 3.69 MPa, 2.23 MPa, 2.06 MPa, and 1.39 MPa, respectively. Therefore, the tensile strength test shows that the composite material after being alkali-treated has the best tensile strength, followed by the composite material after being acid-treated, the composite material after wet–heat treatment and the composite material without treatment.

Figure 6b shows the image of the composite material when it fractures in the tensile test. It can be seen from the figure that the fracture direction is along the width direction of the composite material and the fracture point is the middle part of the fracture, which excludes the factor of material fracture caused by over-tightening of the fixture.

Chikesh Ranjan’s team in India [40] reached the same conclusion when using the same method to prepare composites. The results show that pretreatment can remove silica and lignin on the surface of straw, and straw pretreatment can improve the tensile properties of composite materials. Furthermore, tensile strength increased from 2 MPa to 14 MPa. In addition, the pretreatment methods used by the researchers were alkali treatment and wet heat treatment, and the concentration of NaOH selected for alkali treatment was 2%. The concentration of NaOH used in this study was 12%, so the pretreatment effect in this study was more significant than that used by the researchers. The straw used by the researchers is a whole stalk, while the straw fiber used in this study is in the range of 250 μm particle size, so there is a significant difference in tensile strength. In addition, the tensile modulus was also analyzed in this study, and the variation obtained was consistent with that of the tensile strength, which fully indicated that the pretreatment effect of this study was better. Although the results of this study are similar to the tensile properties of the materials prepared by the researchers, the reason for this may be the uneven application of the release agent.

### 3.2. Flexural Strength

Table 7 and Table 8 show the ANOVA of the test results of flexural strength and flexural modulus. It can be seen from the ANOVA that the effect of straw fiber filling amount and pretreatment method on flexural strength and flexural modulus is extremely significant, indicating that straw fiber filling amount and pretreatment method have an evident influence on flexural performance.

**Figure 7:**
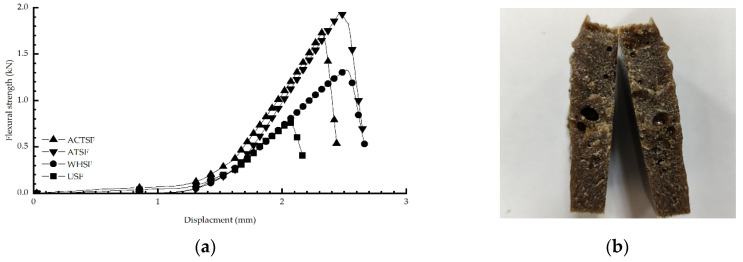
Flexural strength of composite materials: (**a**) the curve of flexural strength; (**b**) composite failure picture.

Figure 7a shows the composite flexural strength versus displacement images, from which it can be seen that the maximum fracture values of the pretreated composites are significantly larger than those of the untreated composites. Moreover, the maximum tensile strength of the alkali-treated composites had the best maximum flexural strength of 2.00 KN. They are followed by the acid-treated composites with a maxi-mum tensile strength of 1.85 KN. Compared with the other two pretreatments, the flexural strength of the moist wet-heat treated composites was not significant with a maximum tensile strength of 1.11 KN. The corresponding tensile stresses of the four pretreatment methods reached 81.65 MPa, 72.77 MPa, 55.78 MPa, 32.52 MPa, and 23.86 MPa, respectively. Therefore, the flexural strength test shows that the composite material after alkali-treated has the best flexural strength, followed by the composite material after acid-treated, the composite material after wet–heat treatment and the composite material without treatment.

Figure 7b shows the image of the composite material when it fractures in the flexural strength. It can be seen from the figure that the fracture direction is along the width direction of the composite material and the fracture point is the middle part of the fracture, which excludes the factor of material fracture caused by over-tightening of the fixture.

It can also be seen from the figure that when the fiber filling amount is 15%, the composite material prepared has the best bending performance, among which the composite material after alkali treatment has the most robust bending performance, with the bending strength and bending modulus of 34.86 and 2191 MPa. The flexural strength and flexural property of the composites without pretreatment are significantly improved by 24.1% and 32.1%, respectively, compared with those without pretreatment, which are 26.47 and 1487 MPa. It can be seen from the trend lines in Figure 7a,b that the bending property of the composites increases first and then decreases, which is consistent with the tensile property. The flexural strength and modulus of the composites after acid treatment are 32.03 and 2016 MPa, which is stronger than that of the untreated composites. Similarly, the damp–heat treatment can improve the bending property of the composites, but the effect is not as significant as that of the alkali treatment. After treatment, the straw fiber will not agglutinate in the preparation of materials; that is, the fiber and epoxy resin are not mixed evenly, and the composite material will not have stress concentration.

Chikesh Ranjan’s team in India [40] found that the bonding effect between rice straw and epoxy resin was poor, so the straw was treated by pretreatment, and the maximum flexural strength and flexural modulus of the treated composite reached 33 MPa and 3700 MPa, respectively. The results are consistent with those obtained in this study. However, the straw fiber composites used in this study are significantly better than the whole rod composites used by the researchers. The straw fiber used in this paper has a remarkable defibrillating effect after pretreatment, so the epoxy resin can be thoroughly combined with RSF and has excellent bending performance.

### 3.3. Water Absorption Test

**Figure 8:**
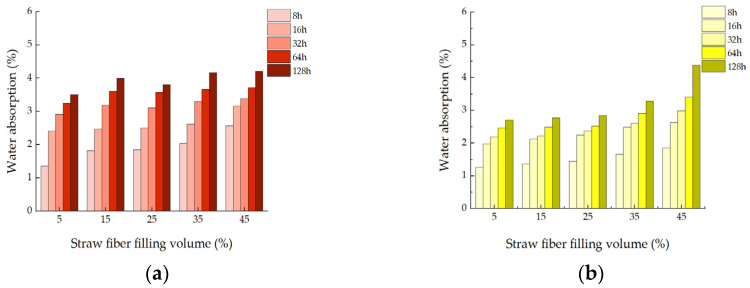

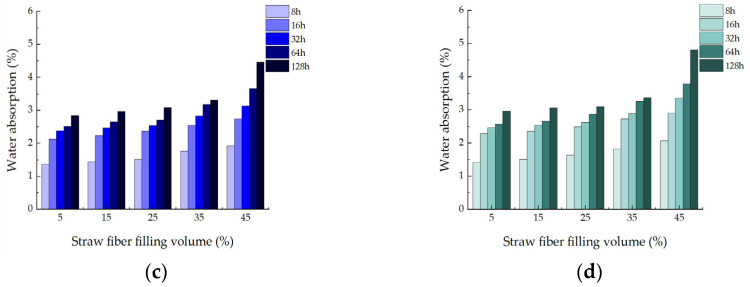
Testing of moisture content of composite materials: (**a**) water absorption of untreated composites with different fiber fillings; (**b**) water absorption of alkali-treated composites with different fiber filling; (**c**) water absorption of acid-treated composites with different fiber fillings; (**d**) water absorption of wet-heat treated composites with different fiber filling capacity.

It can be seen in Figure 8a–d that, for the absorbent composite material under different pretreatment methods, the results showed that the preparation of the composite untreated water absorption performance is high, and with straw fiber filled in the epoxy resin, the water absorption performance of composite materials will also increase; this is because the fiber containing cellulose, hemicellulose, and lignin substances, these substances can have excellent hydrophilicity. In addition, the surface of straw fiber has a large number of hydroxyl groups but also has strong water absorption, which weakens the waterproof of the composite material. Water molecules can seep into the material through tiny gaps between the epoxy layers. The increasing amount of straw fiber makes the barrier effect of the composite material to water worse, and the water absorption rate of the composite material becomes higher and higher.

On the other hand, the water absorption rate of the composite after pretreatment is significantly lower than that of the untreated composite, and the water absorption performance of the composite after alkali treatment is the best. Because the pretreatment removes the impurities on the fiber surface, the alkali treatment dissolves some organic matter such as pectin, lignin and sugar, and the straw fiber surface becomes rough, increasing the internal density and interfacial property of the composite material, and the water absorption gradually decreases. Epoxy resins are hydrophobic materials that can block some water molecules from entering. Moreover, the -OH exposed on the fiber surface can also react with the epoxy group, which reduces the internal porosity of the material and further reduces the water absorption of the composite. After pretreatment, water molecules cannot easily pass through the pores in the material, and the weak interface will not diffuse, accompanied by ceramic powder and magnesium carbonate, to fill the gap and strengthen the material’s hydrophobicity.

**Figure 9:**
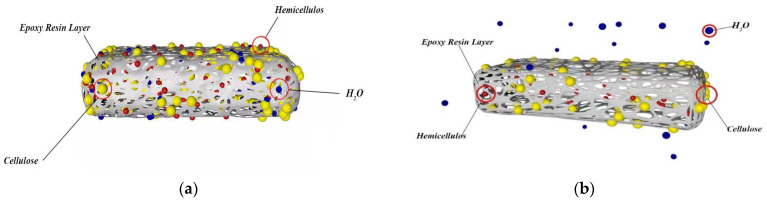
Schematic diagram of water absorption performance of composite materials: (**a**) flat stage; (**b**) secondary ascent stage.

The water absorption rate of composite materials can be divided into three stages: 5–15% rising stage, 15–25% gentle stage, and 25–45% rising stage. From the overall trend, the composites’ water absorption increases with the filling amount. The specific trends are as follows. For example, the initial increase in untreated composite materials is that numerous pores will be formed between fibers and epoxy groups during the molding process of composite materials, and water molecules will enter into the composite materials along these pores, increasing the water absorption rate of composite materials. As shown in Figure 9a, the flat stage is because the epoxy resin is a three-dimensional network structure, and a protective film (epoxy resin layer) will be formed on the material’s surface to inhibit water intake, so it is not easy for water molecules to enter. Especially after pretreatment, cellulose and hemicellulose have a bonding effect, forming a bridge between straw and epoxy resin. Therefore, the interface bonding performance of composite materials is enhanced, and the intermolecular interval becomes smaller, which decreases surface porosity. Thus, the change in water absorption at this stage is not apparent. As shown in Figure 9b, the second ascending stage is because, with the increase in fiber filling, the cellulose contained in the fiber is hydrophilic so that it can absorb many molecules of water. In addition, with the reduction in epoxy resin, the material surface cannot provide a better water-inhibiting environment, which leads to improved water also the option performance of the composite. The moisture content of the composite material being too high will affect the mechanical properties of composite material. It cannot form a stable structure, so it is crucial to select the appropriate amount of filling.

Mittal, V and Sinha, S [41] mainly explored the preparation of composite materials jointly with epoxy resin after wheat straw pretreatment. The researchers used different concentrations of NaOH to treat straw and found that pretreatment significantly improved the water absorption performance of composite materials, which was consistent with the research results in this paper. However, researchers found that straw fiber filling reduced the mechanical properties of the composites, which may be related to incomplete alkali treatment. In this study, 12% NaOH was used for treatment, which can make the straw fiber more thoroughly remove the surface impurities and combine it with epoxy resin.

### 3.4. Density Determination

**Figure 10:**
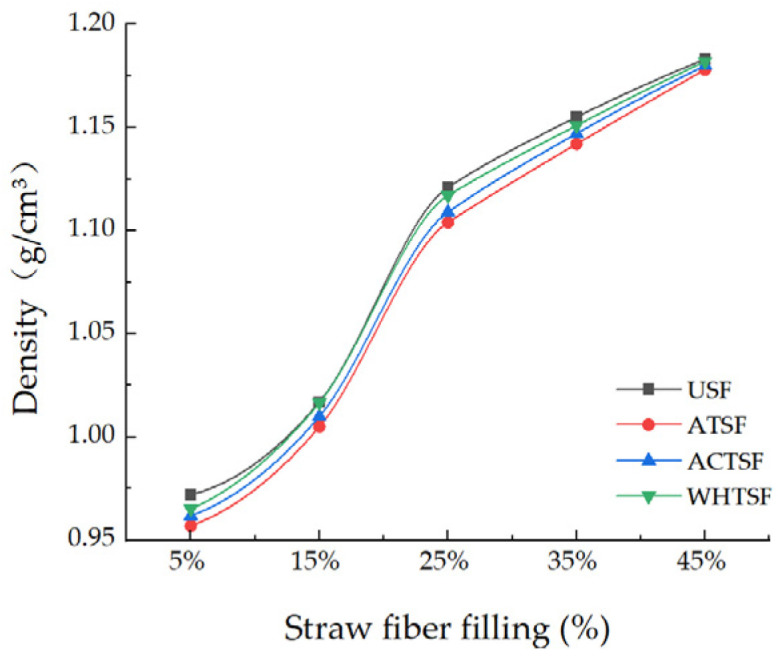
Relationship between density and fiber volume fraction of composites by different pretreatment methods.

Figure 10 shows the density of composite materials under different straw fiber pretreatment. It can be seen from the table that the density of composite materials after pretreatment is significantly lower than that of composite materials without treatment. Additionally, the composite after alkali treatment showed a better reduction. This is because the surface of untreated straw will have uneven waxes, affecting the chemical interaction between cellulose and epoxy groups [42]. The cellulose in the straw cannot be closely combined with the epoxy resin, and the interface bonding effect with the epoxy resin is not good. Significant gaps and holes in the straw lead to the phenomenon of high density. In addition, due to the internal pores, when the material is stressed, it cannot extend from the epoxy matrix to the straw fiber, which significantly reduces the mechanical properties of the composite. The figure also shows that under the same pretreatment method, the changing trend of composite density increases with the straw fiber filling amount. When the filling amount of straw fiber is 10–15%, the density of the prepared composite material reaches the minimum. In general, for different pretreatment methods, other conditions were the same, when the straw fiber filling amount reached 45%, the composite density reached the maximum.

**Figure 11:**
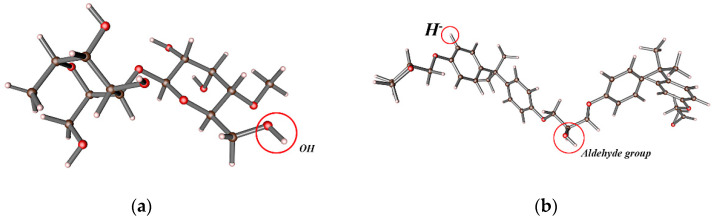
Cellulose and epoxy ball and stick model: (**a**) cellulose ball and stick model; (**b**) epoxy ball and stick model.

Under the same straw fiber filling amount, the changing trend of composite density after alkali treatment is more pronounced. Because the treated straw fiber has a rough surface and a large amount of -OH is exposed, as shown in Figure 11, which is easy to bond with the epoxy surface due to chemical interaction, resulting in mechanical interlocking between the two and reducing the internal pore structure. After alkali treatment, the surface of the wet straw fiber is rougher, which improves the mechanical interlocking strength with the epoxy base and enhances the adhesion between straw fiber and matrix so that the physical and mechanical properties of the composite material are excellent. When the filling amount of straw fiber is small, the fiber can be well mixed with the epoxy group to toughen. When the fiber content of straw increases to 15%, the mixing state with polymer reaches the best, and the interface contact between them is good [20]. Due to the tight density, the mechanical properties of the composite are better, the stress can be well transferred from the polymer to the straw fiber, the interfacial transfer efficiency is improved, and the mechanical properties of the composite are further enhanced. As shown in the figure in the binding process of straw fiber and epoxy resin, there are active functional groups such as hydration (H-) and aldehyde groups on the epoxy resin interface, which can form a network structure with cellulose and hemicellulose in the fiber. A small number of pores will also be generated in forming the network. Adding ceramic micro-powder and magnesium carbonate will enter the resulting pores along with straw fiber in the preparation process, reducing the density of composite material to a certain extent.

Zhang, L et al. [43] found that straw reinforced low-density polyethylene composites with straw must be processed into fiber or powder, which is more conducive to enhancing the properties of the materials, and it is easy to combine with urea-formaldehyde resin to improve the properties of the composites. This research result is consistent with this paper. The researchers found that the composite material prepared with straw fiber had the best density, and the optimal density ranged from 0.83–1.31 g/cm^3^, similar to this paper’s research results. However, the minimum density of the composite material in this paper is too high, which may be caused by the researchers’ remarkable compaction effect of the hot-pressing technology.

### 3.5. Surface Aperture Analysis

**Figure 12:**
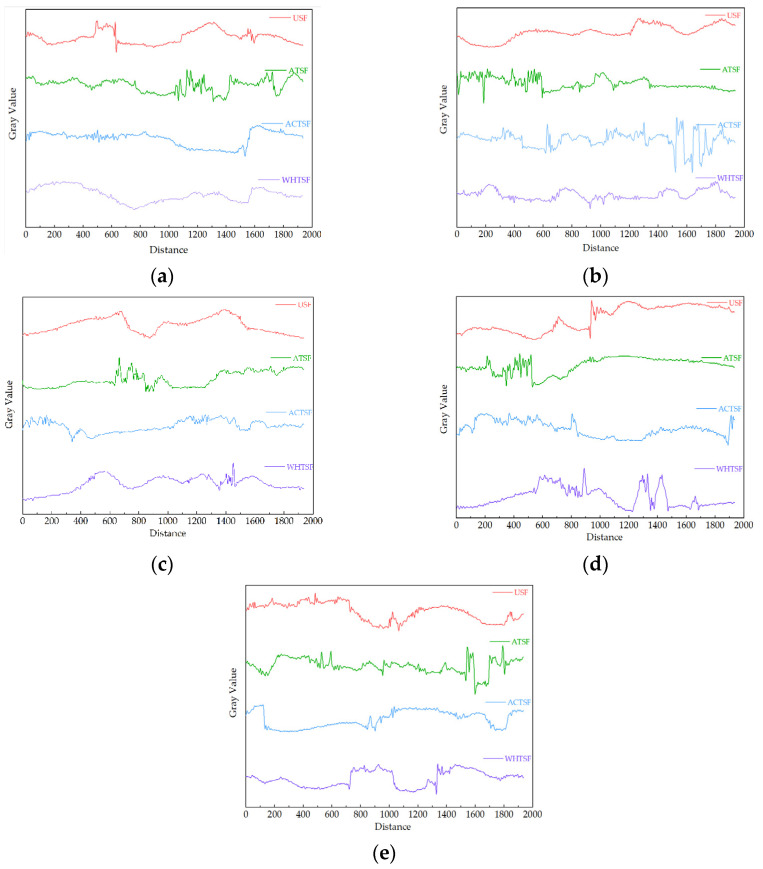
Effect of pretreatment methods on pore size of straw composite under different straw fiber filling: (**a**) 5%; (**b**) 15%; (**c**); 25%; (**d**) 35%; (**e**) 45%.

Figure 12 shows the pore size diagram of straw fiber composite material with different filling quantities under different pretreatments. It can be seen from the figure that the wave peaks in the image after pretreatment are significantly more frequent than those of USF, indicating that the internal pores of the composite material are widely distributed, the overall permeability of the composite material is strong, and the composite material has more excellent adsorption performance. It can also be seen from the figure that the pore size of straw composite material prepared after alkali treatment of straw fiber is significantly larger than that of other pretreatment methods because the surface wax of straw fiber after alkali treatment is removed, which can provide a large amount of OH^-^ to combine with an epoxy group. Since straw fiber is an irregular structure and epoxy resin is a three-dimensional structure, the epoxy resin will tightly surround the straw fiber in the binding process, producing many pores. The pore size under other pretreatment methods is too small, possibly because the straw fiber has less interface available for the binding of epoxy resin, and the epoxy resin is in an unbound state. Due to the viscous shape of the epoxy resin, there will be a specific connection between each other and mutual bonding, resulting in too few pores of the composite material.

The grayscale range in the figure is 20–220. It can be seen that when the filling amount of straw fiber is 15%, the composite material has more wave peaks, that is, more pore size, so the composite material shows better performance. Compared with other filling quantities, the straw fiber at 15% can provide more OH^-^, the interface area available for epoxy resin bonding is more significant, and more pores are realized.

To sum up, when the straw fiber filling amount is 15%, the method of alkali treatment can make the straw composite material show better pore structure, and the composite material can show better adsorption performance and light texture, without any volatile substances, and meet the basic requirements of high density fiberboard, in line with the contemporary advocacy of green environmental protection this theme.

This section may be divided into subheadings. It should provide a concise and precise description of the experimental results, their interpretation, and the practical conclusions that can be drawn.

### 3.6. Discussion

Currently, the primary method of preparing straw fiber and epoxy resin composites is to prepare straw with palm, bamboo, coconut shell, and other materials, and then with polyethylene, polyvinyl chloride, and other materials. The preparation process is not only complicated but also causes environmental pollution to the environment. As far as we know, now the rice straw fiber and epoxy resin, respectively, used in straw composite materials research, but will use a combination of study is less, the comprehensive testing of the straw composites research is less. Hence, the analysis of innovative rice straw fiber composites also adopted a new method to compare the pore structure of composites, and the results are scientifically verified.

Xuan et al. [43] discussed the influence of straw-based wood-based panel performance by modifying straw surfaces in the form of KH550, KH560, and heating in the experiment. As for straw pretreatment in this study, the surface of straw exposed OH- increased after straw treatment, which can improve the physical and chemical properties of the composite. The difference is that in the preparation of composite materials in this study, straw is used to remove ash and wax on the surface of straw with chemical reagents before use, which can obtain purer straw fiber. Compared with the organic solvent method, the extract in this study is purer and safer to operate. The adhesive used in straw-based wood-based panels is urea-formaldehyde resin mixed 1:1 with water. The urea-formaldehyde resin has disadvantages such as long curing time, incomplete curing, and poor bonding quality. Therefore, the method of cross-linking agent is adopted to modify the urea-formaldehyde resin further. The epoxy resin used in this study has a high viscosity, strong adhesion, slightly longer curing time, and better curing effect, but also has zero volatile organic compounds, an environmentally friendly material. Straw-based wood-based panels are mainly preprocessed with urea-formaldehyde resin through a cross-linking agent, only the mechanical properties of the composite materials are tested, and the chemical properties such as oil resistance and corrosion resistance are not mentioned. In this study, the OH^-^ was exposed after straw pretreatment was combined with the epoxy group in epoxy resin to form a stable three-dimensional network structure. Straw-based wood-based panel adopts a hot-pressing method to create the board, which is easy to operate, but the process is complex. The molding method adopted in this study is casting, and the casting model is not limited in shape and thickness. Compared with the straw-based wood-based board, the board in this study can choose the mold according to the application situation, which is more convenient and flexible.

## 4. Conclusions

Epoxy resin is used in aerospace, marine and construction because of its corrosion resistance, excellent mechanical properties, and stable chemical properties, among which construction is mainly made into plates for broad applications. Straw fiber as an alternative reinforcement material for reinforced epoxy resin composites has attracted the attention of scholars and has become one of the most widely researched directions in recent years. The mechanical properties of the composites have been tested and characterized as a significant indicator of the performance of the composites. This study prepared a new composite material using rice straw fiber-reinforced epoxy resin. The principle is to use the cellulose and other components exposed by the straw after pretreatment can be combined with the epoxy group. The best pretreatment method was derived by observing the fiber unfibrillation effect of the straw fiber pretreatment method. Additionally, the combined effect of the best pretreatment method and straw fiber filling on the performance of the composite was verified, and the following conclusions were drawn:

- Through the characterization analysis of the pretreatment straw fiber and the control group (untreated rice straw fiber), it was found that the straw fiber after alkali treatment produced the most “silk”, the best defibrillation effect. Secondly, straw fiber after acid treatment; The defibrillation effect of the straw fiber after wet-heat treatment is poor.
- Compared with the control group, it was found that under the same pretreatment method, the composite’s tensile strength, tensile modulus, flexural strength, and bending modulus reached1.89 KN and 3.92 MPa, 2.00 KN and 81.65 MPa, respectively, when the straw fiber filling amount reached 15%. When the same amount of straw fiber is filled, the composite material after alkali treatment has the best performance, followed by the composite material after acid treatment, and the composite material after wet and hot treatment has poor performance.
- In the water absorption test, the change process of the composite material is divided into two stages, namely the rising stage and the continuous rising stage. The water absorption performance of the composite mainly depends on the rising stage. When the filling amount of straw fiber reaches 15% and the water absorption time is 128 h, the performance of the composite reaches its best. The optimal water absorption rate of the composite material after alkali treatment reached 2.77%, the optimal water absorption rate of the composite material after acid treatment reached 2.97%, and the optimal water absorption rate of the composite material after wet and hot treatment reached 4.81%.
- In the density test of composite materials, when the straw fiber filling amount reaches 5%, the density of composite materials is the minimum. Among them, the minimum density of the composite material after alkali treatment is 0.96 g/cm^3^, the minimum density of the composite material after acid treatment is 0.961 g/cm^3^, and the minimum density of the composite material after wet and hot treatment is 0.965 g/cm^3^.
- The pore structure and distribution of composite materials were further verified by Image J software. The results showed that when the straw fiber filling amount was 15%, the composite materials prepared by the alkali treatment method had the smallest pores and minor distribution.

In conclusion, when the alkali treatment method is adopted, the performance of the composite material is the best when the straw fiber filling amount is 15%, and the composite material meets the basic requirements of high-density fiberboard.

This paper can provide a reference for preparing straw composites, especially the surface pore analysis of the composites. However, since it was not compared with other straw fibers, such as sorghum, bamboo, and soybean, subsequent studies are needed to compare with other composites prepared from straw. In addition, the composites in this study have slightly more surface pores, which is the main reason for the lack of mechanical properties. Therefore, it should also be explored how to reduce the surface porosity of the composites to improve the composite properties. This paper can provide a reference for the preparation of straw composites, especially the analysis of surface pores of composites. However, since it was not compared with other straw fibers, such as sorghum, bamboo, and soybean, subsequent studies are needed to compare with other composites prepared from straw. In addition, the composites in this study have slightly more surface pores, which is the main reason for the lack of mechanical properties. Therefore, it should also be explored how to reduce the surface porosity of the composites to improve the composite properties.

## Figures and Tables

**Table 1:** Group chemical composition of straw from different straw crops [30].

Straw Type	Dry Matter (%)	Ash (%DM)	Crude Protein (%DM)	Fiber Composition (%)		
				Fiber	Hemicellulose	Lignin
Rice straw Maize straw Wheat straw Sorghum straw	95.04 96.08 91.02 93.47	19.43 6.98 6.37 6.03	3.19 3.24 2.57 3.39	39.63 32.92 38.09 42.23	35.50 32.48 34.03 31.58	13.92 4.63 14.13 7.62

**Table 2:** The range of chemical composition of rice straw cell walls [30].

Constituent (%)	Rice Straw			
	Husk	Whole Straw	Leaf	Stem
Fiber Hemicellulose Lignin Residual ash	36–43 19–25 18 12–16	45–60 33 8–19 6–33	37–41 22–25 7–8 29–37	23–44 24–28 4–6 8–16

**Table 3:** Test materials.

Material	Manufacturer
epoxy resin	Shenyang Dongyan Paint Decoration Co., LTD, Shenyang City, Liaoning Province, China
curing agent	Shenyang Dongyan Paint Decoration Co., LTD, Shenyang City, Liaoning Province, China
rice straw	Experimental field, Shenyang Agricultural University, Liaoning Province, China
ceramic powder	Shijiazhuang City, Hebei Province, China Lingshou County Chuangkai Mineral Products Co., LTD
magnesium carbonate	Hebei Songzhi Chemical Technology Co., LTD. Shijiazhuang City, Hebei Province, China 193.5195
magnesium sulfate	Tianjin Zhiyuan Chemical Reagent Co., LTD
mold release	China Suzhou Kangxing Chemical Technology Co., LTD
antifoaming agents	China Shandong Xinrunjin Chemical Co. LTD
sodium hydroxide (NaOH)	analytically pure
oxalic acid (HOOCCOOH)	analytically pure

**Table 4:** Test instruments.

Instrument	Manufacturer
straw silk kneading machine	Shenyang Agricultural University, Shenyang City, Liaoning Province, China
straw fiber crusher	Shenyang Agricultural University, Shenyang City, Liaoning Province, China
far-infrared ray drying oven	Tianjin Tongli Xinda Instrument Factory, China
circulating water vacuum pump	Zhengzhou, Henan, China Zhengzhou Yuda Instrument Technology Co., LTD
high-speed crusher	Shanghai, China Dingshuai Electric Appliance Co., LTD
water bath kettle	Shanghai, China Lichen Instrument Technology Co., LTD
high-speed blunger	Shanghai, C hina Meiyingpu Instrument Manufacturing Co. LTD
ultra-depth of field microscope (VHX-5000)	Keansy(China),Shanghai City, China, Co., LTD
universal testing machine	Instrong(China),Shanghai, City, China, Co., LTD
silastic mold	Henan Pingdingshan Xingyucheng Mould Factory, Henan city, China

**Table 5:** ANOVA of tensile strength.

Source of Variance	SS	df	MS	F	*p*-Value
row line error value total	12.45905 175.2678 5.79263 193.5195	11 4 44 59	1.132640455 43.8169525 0.131650682	8.60337705 332.8273876	7.56 × 10 ^−8^ 2.88 × 10 ^−32^

**Table 6:** ANOVA of tensile modulus.

Source of Variance	SS	df	MS	F	*p*-Value
row line error value total	267638.5333 2510385.167 96851.63333 2874875.333	11 4 44 59	24330.77576 627596.2917 2201.173485	11.05354754 285.1189586	2.06 × 10 ^−9^ 7.66 × 10 ^−31^

**Table 7:** ANOVA of flexural strength.

Source of Variance	SS	df	MS	F	*p*-Value
row line error value total	540.4041783 235.40791 104.33813 880.1502183	11 4 44 59	49.12765258 58.8519775 2.371321136	20.71741858 24.81822331	6.61411 × 10^−14^ 8.53167 × 10^−11^

**Table 8:** ANOVA of bending modulus.

Source of Variance	SS	df	MS	F	*p*-Value
row line error value total	4924897.65 4254198.167 415449.4333 9594545.25	11 4 44 59	447717.9682 1063549.542 9442.032576	47.41754115 112.6398933	9.98757 × 10^−21^ 1.60787 × 10^−22^

## Data Availability

The data presented in this study are available on request from the corresponding author.
